# Fusion protein-based COVID-19 vaccines exemplified by a chimeric vaccine based on a single fusion protein (W-PreS-O)

**DOI:** 10.3389/fimmu.2025.1452814

**Published:** 2025-01-28

**Authors:** Pia Gattinger, Luibov I. Kozlovskaya, Alexander S. Lunin, Olga S. Gancharova, Dina I. Sirazova, Vasiliy D. Apolokhov, Egor S. Chekina, Ilya V. Gordeychuk, Alexander V. Karaulov, Rudolf Valenta, Aydar A. Ishmukhametov

**Affiliations:** ^1^ Center for Pathophysiology, Infectiology and Immunology, Department of Pathophysiology and Allergy Research, Division of Immunopathology, Medical University of Vienna, Vienna, Austria; ^2^ Chumakov Federal Scientific Center for Research and Development of Immune-and-Biological Products of Russian Academy of Sciences (Institute of Poliomyelitis), Moscow, Russia; ^3^ Institute for Translational Medicine and Biotechnology, Sechenov First Moscow State Medical University, Moscow, Russia; ^4^ Laboratory for Immunopathology, Department of Clinical Immunology and Allergology, Sechenov First Moscow State Medical University, Moscow, Russia; ^5^ Life Improvement by Future Technologies (LIFT) Center, Moscow, Russia; ^6^ Center for Molecular Allergology, Karl Landsteiner University of Health Sciences, Krems, Austria

**Keywords:** SARS-CoV-2, COVID-19, omicron, vaccine, fusion protein-based vaccine, neutralizing antibodies, infection model, Syrian hamster

## Abstract

In this article we discuss characteristics of fusion protein-based SARS-CoV-2 vaccines. We focus on recombinant vaccine antigens comprising fusion proteins consisting of combinations of SARS-CoV-2-derived antigens or peptides or combinations of SARS-CoV-2 antigens/peptides with SARS-CoV-2-unrelated proteins/peptides. These fusion proteins are made to increase the immunogenicity of the vaccine antigens and/or to enable special targeting of the immune system. The protein-based vaccine approach is exemplified solely in a proof of concept study by using W-PreS-O, a chimeric vaccine based on a single fusion protein (W-PreS-O), combining RBDs from Wuhan hu-1 wild-type and Omicron BA.1 with the hepatitis B virus (HBV)-derived PreS surface antigen adsorbed to aluminum hydroxide. The W-PreS-O vaccine was evaluated in Syrian hamsters which were immunized three times at three-week intervals with W-PreS-O or with aluminum hydroxide (placebo) before they were infected with Omicron BA.1. Neutralizing antibody (nAB) titers, weight, lung symptoms, and viral loads, as measured using RT-PCR in the upper and lower respiratory tracts, were determined. In addition, infectious virus titers from the lungs were measured using a plaque-forming assay. We found that W-PreS-O-vaccinated hamsters developed robust nABs against Omicron BA.1, showed almost no development of pneumonia, and had significantly reduced infectious virus titers in the lungs. Importantly, the viral loads in the nasal cavities of W-PreS-O-vaccinated hamsters were close to or above the PCR cycle threshold considered to be non-infectious. The data of our proof-of-concept study provides compelling evidence that the W-PreS-O vaccine has protective effect against Omicron BA.1 in a Syrian hamster *in vivo* infection model and thus support the promising results obtained also for other fusion protein-based SARS-CoV-2 vaccines.

## 1 Introduction

The ongoing Severe acute respiratory syndrome corona virus-2 (SARS-CoV-2), the causative agent of COVID-19, became endemic approximately four years after its pandemic outbreak in late 2019 (1–3). At present, the severity of COVID-19 symptoms in the general population is lower than at the beginning of the pandemic (4, 5). However, COVID-19 remains a major health issue, and it can present with high disease severity and mortality in vulnerable patient groups (6–8). At least two mutually non-exclusive explanations for the lower severity of COVID-19 in the general population may be considered. One possibility is that pre-existing immunity caused by previous infections or vaccinations has been established and mitigates recurrent infections and disease severity (9, 10). The other explanation for the lower severity and mortality of COVID-19 is that the currently predominating Omicron variants are less pathogenic (11–13). The SARS-CoV-2 variant B.1.1.529, now termed Omicron, was announced by the World Health Organization on November 24, 2021, and it appears to have an increased risk of infectivity. A recent meta-analysis of reports regarding disease severity concluded that the mortality rates of patients infected by Omicron ranges from 0.01% to 13.1%, while in patients infected with previous variants it was 0.08% to 29.1% (14). Thus, the mortality rates of Omicron-induced COVID-19 seem to be at least 50% lower than for COVID-19 induced by previous variants. Nevertheless, Omicron-induced infections remain an important problem, and higher infectivity has been reported for Omicron (12, 15, 16). Furthermore, SARS-CoV-2 Omicron can easily escape the immunity established by infection with previous variants and vaccines based on previous SARS-CoV-2 variants (17–20).

Accordingly, the development of vaccines for Omicron and its currently dominating and closely related sub-variants is of high priority (21–24). Alongside the induction of cellular immune responses, like cytotoxic T cells that kill infected cells, it has become clear that antibodies specific for the spike protein S of SARS-CoV-2, and especially against its RBD, are important for protection (25, 26). However, protection requires not only high levels of specific antibodies but also a sufficient breadth of antibody response and, in particular, a high virus-neutralizing capacity.

In this article, we discuss fusion protein-based vaccines for SARS-CoV-2 and highlight some of their features. One type of the fusion protein-based vaccines has the goal to increase the immunogenicity of the vaccines by fusing SARS-CoV-2 antigens/peptides to SARS-CoV-2-unrelated antigens or peptides. For example, it was found that fusion proteins consisting of the S1 part of the SARS-CoV-2 spike protein or the receptor-binding domain (RBD) with the Fc portion of immunoglobulins can enhance the antibody response against the SARS-CoV-2-derived antigens (27–35) Besides increasing immunogenicity, this approach may have also considered targeting Fcgamma-receptor-bearing cells and has progressed towards clinical studies (36, 37). Also other antigens/peptides, for example a tetanus toxoid peptide (38), the Rotavirus-derived VP6 protein (39) and influenza hemagglutinin (40) have been fused to RBD to enhance its immunogenicity. Another type of fusion proteins has been designed to increase mucosal immune responses. For this purpose, RBD has been fused with Salmonella-derived flagellin (41), with *E. coli* heat-labile enterotoxin (42, 43) or with the cholera toxin B subunit (CTB) (44). Fusion proteins were also made to target RBD to antigen presenting cells, for example by fusing it to a MHC II-specific nanobody (45), to MIP3 (46) or to CD154 (47). Yet another type of fusion proteins has been made to broaden the SARS-CoV-2 immune responses. A fusion protein comprising RBD and the nucleocapsid protein (N) (48, 49), RBD and elements of the membrane protein (M) and the N protein (50), RBD heterodimers from different strains (51, 52), a S-trimer (53, 54), a RBD dimer fused to the S-derived N-terminal domain (NTD) (55), a modular train model comprising different RBDs (“cars”) fused to Wuhan S1 protein (“engine”) and a fusion protein containing two immunogenic portions of N fused to S2 (56, 57) fall into this category.

We have developed a fusion protein consisting of the HBV surface antigen PreS flanked by a N-terminal and C-terminal RBD (W-PreS-W) and showed that it is superior in inducing SARS-CoV-2 neutralizing antibodies as compared to RBD alone and in addition, it induces HBV neutralizing antibodies (58). We then further improved this fusion protein-based vaccine by engineering a heterodimeric variant (W-PreS-O) containing one RBD from the original Wuhan strain combined with Omicron-derived RBD. W-PreS-O induced a broadly protective immune response and induced Omicron-neutralizing antibodies better than a fusion protein containing two RBDs from Omicron (O-PreS-O) (24). Here we report results showing that the W-PreS-O vaccine has protective effects against Omicron BA.1 in a Syrian hamster *in vivo* infection model supporting the concept of fusion protein-based SARS-CoV-2 vaccines for the W-PreS-O example.

## 2 PreS-based fusion proteins, an example for fusion protein based COVID-19 vaccines

### 2.1 Design of PreS fusion proteins

Previously, we reported on the design and characterization of a SARS-CoV-2 subunit vaccine candidate based on a fusion protein of two RBDs fused to the hepatitis B (HBV) surface antigen PreS (58). PreS, which comprises preS1 and preS2 of the large hepatitis B virus envelope protein (LHB), has been used as a carrier protein to enhance the immunogenicity of hypoallergenic allergen peptides used in recombinant allergy vaccines (59). In the aforementioned SARS-CoV-2 vaccine, PreS was also used to enhance the immunogenicity of the PreS-fused RBD domains. Since PreS contains the binding site of HBV to its receptor, the sodium-taurocholate co-transporting polypeptide (NTCP), on liver cells. Accordingly PreS-containing vaccines induce antibodies that can protect against HBV infections (60). Another advantage is that PreS-containing fusion proteins are well defined and suitable for reproducible manufacturing according to good manufacture practice (GMP) whereas chemical conjugation to KLH or tetanus toxoid yield relatively undefined products which are difficult to standardize. Finally, recombinant expression is relatively inexpensive and can be easily scaled up to obtain large amounts of the vaccine antigen. The PreS-based SARS-CoV-2 vaccine has been recently further developed and compared with vaccines containing fusion proteins of two RBDs from Omicron fused to PreS, and one chimeric vaccine containing a fusion protein consisting of one RBD from Wuhan and one from Omicron, termed W-PreS-O (24). While all vaccines tested in the latter study induced comparable RBD Wuhan and RBD Omicron-specific antibody levels, the chimeric W-PreS-O shown in **Supplementary Figure S1A** showed a superior capacity to induce Omicron-neutralizing antibodies (24). In fact, W-PreS-O induced 7-fold higher virus-neutralizing titers (VNTs) than the wild-type-specific vaccine (e.g., W-PreS-W) and 2-fold higher VNTs to Omicron than the Omicron-specific vaccine candidate (e.g., O-PreS-O). In this study, we investigated W-PreS-O, shows protective effects *in vivo* against SARS-CoV-2 Omicron infections in Syrian hamsters.

### 2.2 Immunization with W-PreS-O induces SARS-CoV-2 Omicron BA.1-neutralizing antibody titers in Syrian hamsters

Materials and methods supporting the data in sections 3.1 – 3.4 can be found in the Supplement to this article. The design for the Syrian Hamster study can be found in **Supplementary Figure S1B**. **Supplementary Figures S2**-**S4** show the amino acid sequence of the W-PreS-O vaccine antigen, that the O-RBD sequence of the vaccine is almost identical with the RBD sequence of the BA.1-like sub-variant strain 7955o used for infection in our study and the high sequence similarity of the RBD from the BA.1 variant in the vaccine construct with more recently described Omicron variants, respectively. In fact, the most recent Omicron variants JN.1, KP.2 and KP.3 differ in the RBD sequence from that used in our W-PreS-O vaccine and BA.1 strain used in our infection model only by four amino acids of which only three exchanges are not conserved (**Supplementary Figure S4**).

**Figure 1:**
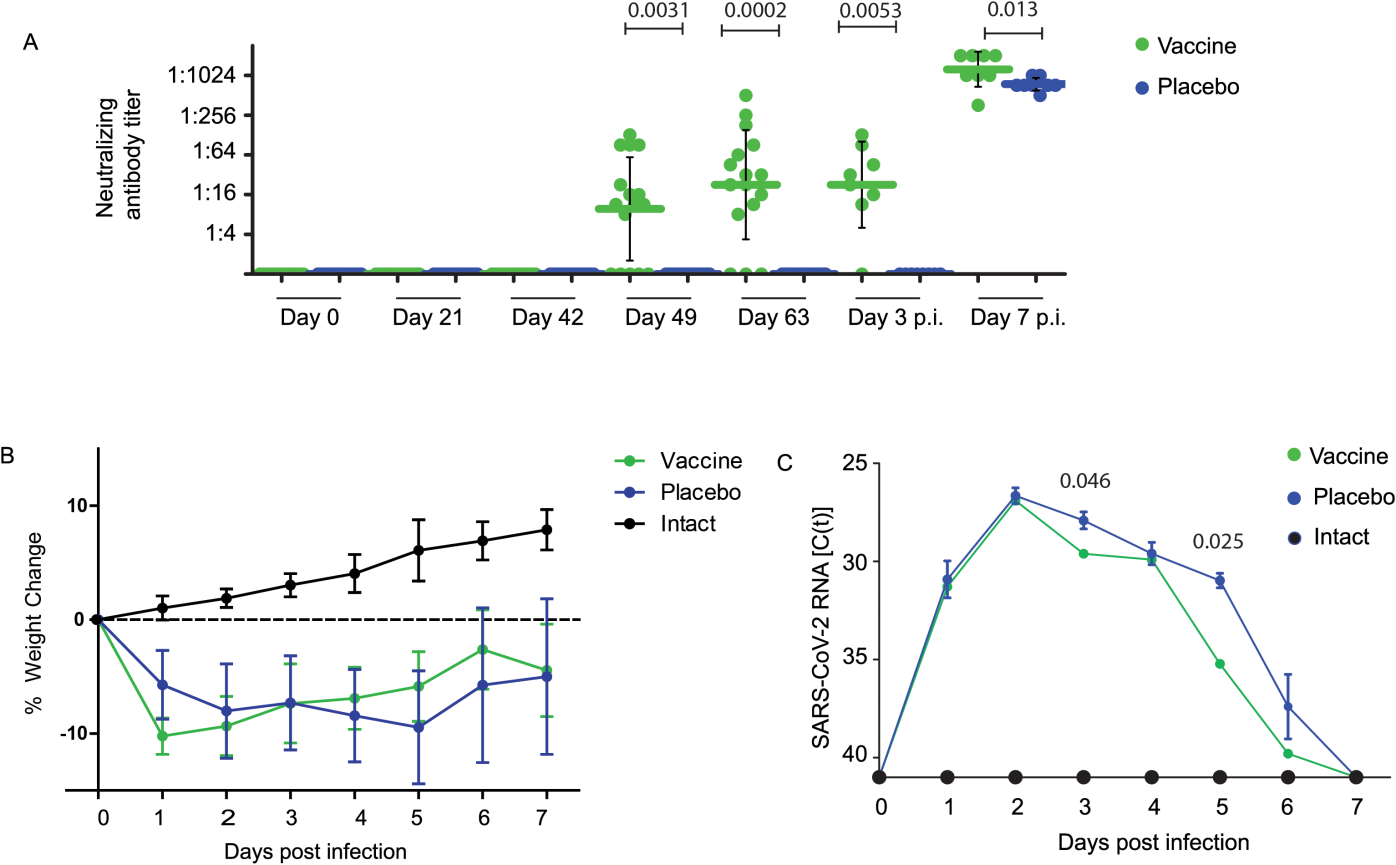
Neutralizing antibodies, body weight, and viral RNA load in oropharyngeal samples in Syrian hamsters. **(A)** Neutralizing antibody titers for the SARS-CoV-2 variant Omicron in sera (shown as a serum dilution, y-axis) were obtained at the indicated time points during immunization and after infection with virus. Titers below 1:8 were considered negative. Horizontal bars represent GMT values for each group (Green: vaccinated; blue: placebo; black: not infected=intact). The dots are results for each animal and the whiskers indicate standard deviations. Significant differences (p < 0.05) were determined with the Mann–Whitney test and are indicated. **(B)** Weight curves are presented as percentages of body weight change (y-axis) and **(C)** viral RNA contents in oropharyngeal swabs as cycle threshold C(t) values (y-axis) on the indicated days post-infection. C(t) values >40 were considered negative. The results are shown as mean values per group and standard deviations. Significant differences (p < 0.05) were determined and are indicated at observed time points.

**Figure 1A** demonstrates that the geometric mean titers (GMTs) of SARS-CoV-2 Omicron BA.1-neutralizing antibody (nAb) titers induced by vaccination with W-PreS-O increased to mean nAB titers of 23 (median: 27, min: 0, max: 512) in samples obtained on day 63, three weeks after the third immunization (**Figure 1A**). No nABs were detected in sera from the placebo-treated animals (**Figure 1A**) or in sera from the “intact group” (**Supplementary Table S1**) until day 63. On day 63, the animals were challenged intra-nasally with the SARS-CoV-2 Omicron BA.1 strain. Three days post-virus inoculation, no relevant further increase in nAB titers in the W-PreS-O vaccine group was observed, with a GMT of 23 (median: 27). At this time point, no nABs were found in the placebo group (**Figure 1A**; **Supplementary Table S1**) or in the intact group (**Supplementary Table S1**). The nAb response in the vaccinated animals was strongly enhanced by the Omicron BA.1 infection. In the vaccinated group, all but one animal, which already had a nAb titer of 512, increased their nAb titers to 1024 or higher, while only two out of eight animals in the placebo group reached a titer of 1024 through the challenge infection seven days after inoculation (**Figure 1A**; **Supplementary Table S1**). Seven days post infection nAB titers were significantly higher (p= 0.013, Mann-Whitney test) in the vaccinated versus non-vaccinated animals (**Figure 1A**). No nABs were found in the intact group, demonstrating that the inoculation of the virus had occurred only in the W-PreS-O and placebo groups (**Supplementary Table S1**).

### 2.3 Recovery after SARS-CoV-2 infection was faster in animals immunized with W-PreS-O than in placebo-treated animals

Infected animals from both groups treated with either W-PreS-O vaccine or placebo showed weight loss after challenge with SARS-CoV-2 in comparison with animals from the intact group (i.e., non-treated and non-infected animals), in the first three days after infection (**Figure 1B**). This difference may be explained by the fact that animals receiving injections were stressed whereas animals from the intact group experienced no stress through injections. Of note, animals vaccinated with W-PreS-O started to gain weight 3 days post-infection, whereas animals from the placebo group started to gain weight later (i.e., 5 days after infection) (**Figure 1B**). The viral load determined by PCR in oropharyngeal swabs in the two groups showed higher median C(t) values for the vaccine versus the placebo group on days 3 and 5 to 6 after infection (**Figure 1C**).

### 2.4 Effects of vaccination with W-PreS-O on viral loads in the upper and lower respiratory tract and presence of infectious virus in the lungs of infected animals

**Figure 2:**
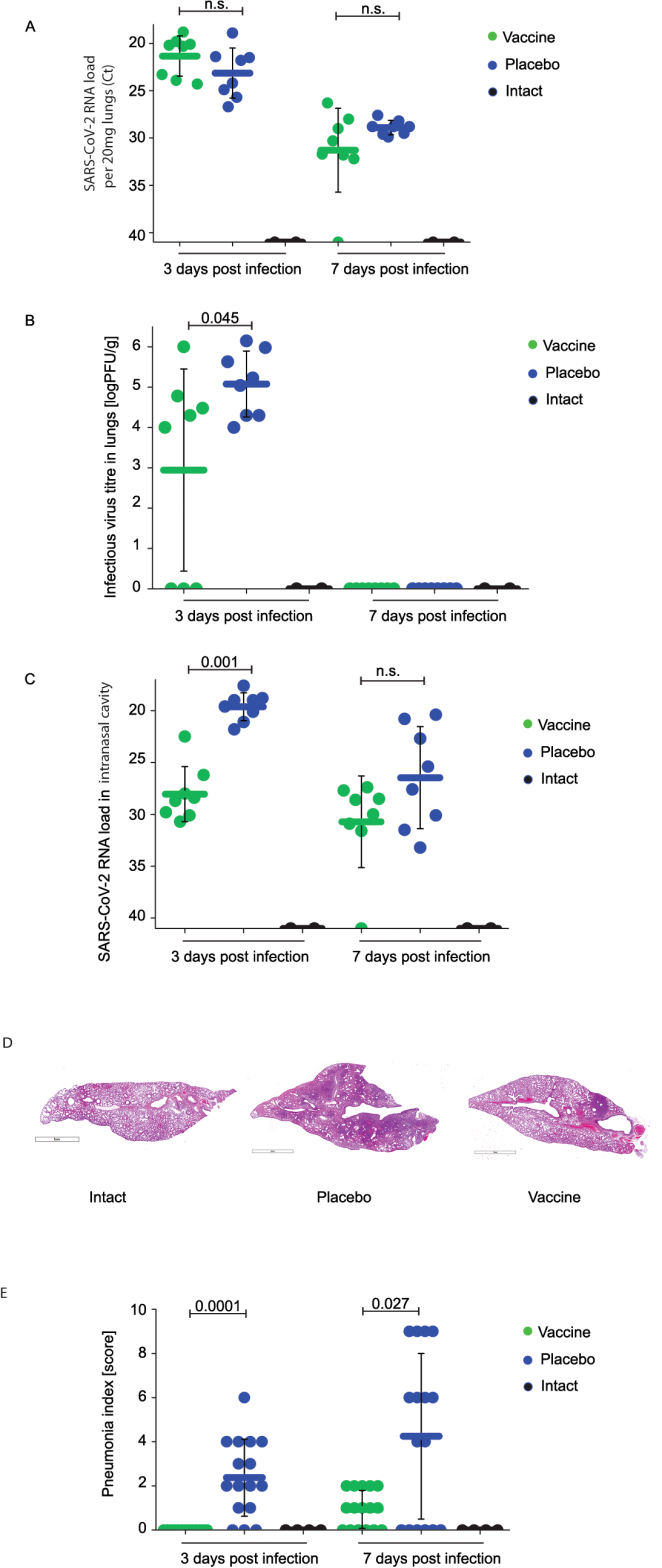
Effect of vaccination with recombinant fusion protein W-PreS-O on SARS-CoV-2 RNA loads in the lungs, infectious virus titers in the lungs, and SARS-CoV-2 RNA loads in the nasal cavities. **(A)** Viral RNA loads and **(B)** infectious virus titers in the lungs or **(C)** viral RNA loads in the nasal cavities of Syrian hamsters (y-axes) immunized three times with the W-PreS-O vaccine (green) or alum (placebo) (blue) at the indicated time points after infection (x-axes) with SARS-CoV-2 variant Omicron. Untreated and non-infected animals (intact) (black) served as controls. Viral loads are given as cycle threshold C(t) values, and were considered negative if >40. Infectious virus titers, determined via plaque titration assay, are presented as logPFU/g values. The results are shown for individual animals, with horizontal bars representing mean values and whiskers indicating standard deviations per group (n = 8). Significant differences (p < 0.05) were determined with the Mann–Whitney test and are indicated. n.s., not significant. Severity of lung lesions in the different groups of infected animals by histological assessment of pneumonia severity is presented in **(D)** as representative microphotographs (lines indicate 3 mm) and **(E)** pneumonia indices (y-axis). The results are shown for individual sections and were calculated from semi-quantitative scores of the lesion area and pneumonia intensity for each section of investigated lung (two sections per animal). Results are shown for individual animals, with horizontal bars representing mean values and whiskers corresponding to standard deviations per group. Significant differences (p < 0.05) were determined with the Mann–Whitney test and are indicated. n.s., not significant.

While there were no significant differences regarding viral loads determined in the lungs of vaccinated and placebo-treated animals by RT-PCR on day 3 and 7 after infection (**Figure 2A**), animals vaccinated with W-PreS-O showed significantly lower infectious virus titers in the lungs (median log PFU/g lung: 4.1, min: 0, max: 6) than the animals immunized with placebo (median log PFU/g lung: 5.1, min: 4, max: 6) three days post-infection (**Figure 2B**). Of note, three out of the eight animals in the vaccine group had cleared the infectious virus from the lung tissue already by day three post-infection (**Figure 2B**). This was in agreement with the finding that on day 3 post-infection, only vaccinated animals, and not placebo-treated animals, had developed nABs (**Figure 1A**). On day seven post-infection, infected animals from both the vaccinated and placebo groups had no infectious virus in the lungs anymore. At this time point, both groups had developed nABs (**Figure 1A**).

Finally, we investigated the viral load in the nasal cavities (i.e., the upper respiratory tract) of the animals (**Figure 2C**). Unlike in the lungs, we found that, on day three after infection, that the animals vaccinated with W-PreS-O had a significantly reduced viral RNA load in the nasal cavities (median C(t) value: 28.6, min: 22.5, max: 30.7) as compared to placebo-immunized animals (median C(t) value: 19.3, min: 17.7, max: 21.8) (**Figure 2C**). This difference was highly significant (p=0.001). Again, this result is consistent with the finding that neutralizing antibodies were present in the vaccinated but not in the placebo group on day 3 (**Figure 1A**). In the W-PreS-O immunized group, two out of eight animals (25%) had C(t) values of 30, and four out of the eight animals (50%) had C(t) values > 28, suggesting that that the animals were not infectious three days post-infection. On day 7 after infection, the mean C(t) value for the vaccinated animals was above 30 cycles, whereas the placebo-treated animals had a mean C(t) value of approximately 27 (**Figure 2C**).

### 2.5 Immunization with W-PreS-O strongly protects against lung damage

Next, we investigated the effects of vaccination with W-PreS-O on Omicron-induced lung damage by comparing pneumonia indices in animals from the vaccine group and placebo group. Untreated and uninfected animals (intact group) served as the controls. In the lung tissue samples of animals from the W-PreS-O immunized group (vaccine) no signs of pneumonia were observed (**Figure 2D**; **Supplementary Table S2**). By contrast, the placebo group animals showed morphological signs of viral pneumonia of varying intensity, from mild bronchitis and incipient pneumonia (bronchopneumonia) to severe viral pneumonia with a characteristic hemorrhagic component and the presence of fibrinous exudate in the alveoli (**Figure 2D**; **Supplementary Table S2**). The lungs from W-PreS-O-vaccinated animals showed some changes of varying intensity at 7 d.p.i., but the severity of these processes was much less pronounced than in the placebo group (**Figure 2E**). In fact, at 3 d.p.i., there was no evidence of pneumonia in the lungs of the W-PreS-O-vaccinated animals, which looked like the lungs from the uninfected hamsters (intact group) (**Figures 2D, E**). Thus, there was a highly significant difference in the pneumonia indices as compared to the placebo-treated animals on day 3 p.i. (p=0.0001) (**Figure 2E**). On day 7 p.i., the pneumonia indices in the W-PreS-O-vaccinated hamsters were very low and the difference, as compared to the placebo group, remained significant (**Figure 2E**, p=0.027).

Edema was estimated as the lung-to-body-weight ratio (**Supplementary Figure S5**). Lung edema was highest in the placebo group 3 and 7 d.p.i. as compared to the W-PreS-O-vaccinated animals and the intact group (**Supplementary Figure S5**).

## 3 Discussion

In this article we have reviewed SARS-CoV-2 vaccines which are based on fusion proteins consisting of SARS-CoV-2-derived proteins or peptides and unrelated proteins or peptides or combinations of SARS-CoV-2 proteins/peptides. These fusion protein-based vaccines have been created with the goal to increase and/or to broaden the SARS-CoV-2-specific immune response and showed promising results in *in vivo* models (27–35, 38, 40, 42–46, 48–53, 55–57, 61–65). For some of the fusion protein-based SARS-CoV-2 vaccines evidence for efficacy has been provided by *in vivo* infection models (30, 34, 49, 51, 53, 62, 64) and for some encouraging data from clinical trials are available (36, 37, 54, 66).

We previously developed a SARS-CoV-2 vaccine based on a recombinant fusion protein consisting of HBV-derived PreS with two flanking RBDs from the Wuhan strain, which induced high levels of neutralizing antibodies against the SARS-CoV-2 variants Alpha to Delta (58). We then refined our vaccine platform for Omicron and could show that a chimeric protein containing one RBD from Wuhan and one from Omicron BA.1 (W-PreS-O) induced a more broadly neutralizing antibody response as compared to a chimeric protein consisting only of two Omicron-derived RBDs (24). Thus, W-PreS-O is a good example of a fusion protein-based SARS-CoV-2 vaccine with enhanced immunogenicity and broadened immune response (24, 58). Furthermore, it seems to have the additional benefit to induce PreS-specific and thus HBV-protective immune responses (58).

We therefore selected W-PreS-O as a candidate vaccine to investigate its ability to protect against Omicron in the Syrian hamster model (24). We found a significant induction of nAB titers in vaccinated animals (**Figure 1**), a significant reduction in infectious virus titers in the lungs in vaccinated versus placebo-treated animals by measuring the infectious virus titers on day 3 (**Figure 2B**). The viral load in the nasal cavity was significantly reduced in vaccinated versus placebo-treated animals on day 3 after infection **Figure 2C**) and importantly, immunization with W-PreS-O significantly reduced lung damage as compared to placebo immunization (see **Figures 2D, E**).

Several additional interesting findings were obtained in the Syrian hamster model when we compared animals vaccinated with W-PreS-O with animals that received placebo. The W-PreS-O-vaccinated animals developed robust nAb responses for Omicron after the last injection as compared to the placebo group. Interestingly, the nAb response in the W-PreS-O-vaccinated animals was strongly enhanced by natural infection with Omicron. In the vaccinated group, all but one animal, which already had a nAb titer of 512, increased their nAb titers to 1024 or higher, while only two out of eight animals in the placebo group reached a titer of 1024 through natural infection by 7 days after inoculation. This might be explained by the fact that vaccination with W-PreS-O had established a broad repertoire of Omicron RBD-specific T cells and B cells, which could be readily boosted by the natural infection. The W-PreS-O vaccine contains a W-PreS-O fusion protein displaying the RBD of Wuhan and Omicron BA.1 as a naturally folded protein mimicking the fold of RBD in the virus (24). The fact that secondary B cell memory in vaccinated animals could be strongly boosted by natural infection suggests that secondary T cell (i.e., CD4^+^ and CD8^+^) responses, which are also critically involved in protection, were eventually also boosted. It is a limitation of our study that non-neutralizing effector functions such as antibody-dependent cellular cytotoxicity (ADCC) or antibody-dependent cellular phagocytosis (ADCP) of the vaccine-induced antibodies versus those induced by infection were not investigated.

W-PreS-O-vaccinated animals showed a faster weight gain and physical recovery from infection than placebo-treated animals as described in other studies performed in Syrian hamsters infected with Omicron (67–69).

The lung is the critical organ in COVID-19 and we therefore carefully studied the protective effects of vaccination with W-PreS-O against Omicron infection in the lower respiratory tract. We observed a significant reduction in the W-PreS-O-vaccinated group as compared to the placebo group on day 3 after virus inoculation regarding infectious virus loads/titers in the lung (**Figure 2B**). The results regarding the significantly reduced infectious virus loads in the lungs can be explained by the presence of nAbs in the vaccinated animals. When nAbs were elevated in the animals, infectious virus titers in the lungs were low (e.g., vaccinated group on day 3 after infection, **Figures 1A**, **2B**). When nAbs exceeded a certain threshold (e.g., vaccinated and placebo 7 days after infection, **Figures 1A**, **2B**), no infectious virus was found in the lungs at all, likely because the nAbs had fully occupied the viral RBDs. The importance of generating nAbs by vaccination with W-PreS-O for protection against lung damage was demonstrated by a detailed histological investigation of the lung tissues (**Figures 2D, E**). No significant edema was found in the W-PreS-O-vaccinated animals as compared to the non-infected (intact) hamsters (**Figures 2D, E**), and there were almost no lung lesions, as demonstrated by the histology and pneumonia indices, in the W-PreS-O-vaccinated animals. In contrast, the placebo-treated animals showed lung edema and elevated pneumonia indices at 3 and 7 days after infection (**Figures 2D, E**).

The Omicron infection model in Syrian hamsters is a complex *in vivo* model that may vary due to many different factors among studies. Nevertheless, studies have been performed with licensed vaccines to study their effect on Omicron infections in Syrian hamsters. Several SARS-CoV-2 vaccines, combinations thereof, and different schedules and dose regimens of vaccination with these vaccines have been tested. It is therefore impossible to compare all these vaccines, combinations, and schedules with our vaccine in one Syrian hamster experiment. Nevertheless, we tried to put our results into the context with other studies by considering infectious viral loads in the lungs because this parameter was assessed in most of the previous Syrian hamster studies. One study was performed with ChAdOx1 nCoV-19 (AZD1222) a replication-deficient simian adenovirus vector-based vaccine encoding S protein from Wuhan-1 and AZD2816 encoding the S protein of SARS-CoV-2 variant of concern Beta. This study showed a reduction of TCID50/g lung tissue as compared to control vector on day 3 but the differences were not significant between vaccine and placebo (70). A study investigating a vaccine based on a recombinant Omicron derived S protein showed a significant reduction of infectious virus in the lungs of Omicron-infected Syrian Hamsters on day 3 compared to placebo similar as we found in our study (67). Another study focused on the possible protective effect of booster vaccinations with licensed mRNA vaccines on Omicron infections in hamsters. This study showed, that two but not one injection showed a modest reduction of infectious virus titers in the lungs (71). Yet another study performing the analysis on days 2 and 4 after infection showed that two to three vaccinations with a heterologous vaccination regimen were able to reduce infectious virus in the lungs as we observed in our study (68). Finally, an interesting result was obtained for CovaxinR, an inactivated SARS-CoV-2 whole virion vaccine which reduced viral load in lung tissue after three doses three days after Hamsters were challenged with BA.2 but no effect for BA.1.1 was observed (72).

It is a limitation of our proof-of-concept study that we have studied the protective effect of our W-PreS-O vaccine only in a hamster model based on Omicron BA.1. We therefore cannot draw firm conclusions that W-PreS-O protects against other Omicron variants. However, a comparison of the amino acid sequences of the RBDs from the BA.1 Omicron variant with more recently described Omicron variants showed only few non-conservative amino acid exchanges (**Supplementary Figure S4**): Three for JN.1, two for BA4/5, six for XBB1.5, six for XBB1.16, one for BA.2, and three for KP.2 and KP.3, respectively (**Supplementary Figure S4**). It is another limitation of our proof of concept study that our W-PreS-O vaccine included the Wuhan variant because since the adaptation to XBB, the recommendations suggest using a monovalent vaccine. In cases where a bivalent vaccine is used, it is suggested not to include the Wuhan variant to evade imprinting effects.

In summary, W-PreS-O seems to be a highly promising SARS-CoV-2 fusion protein-based vaccine which should be further evaluated in clinical trials, once cross-protection to current Omicron variants has been demonstrated by further *in vitro* and *in vivo* experimental data. Such clinical trials should investigate the ability of W-PreS-O to boost a predefined nAb response to currently circulating Omicron variants. Noteworthy, W-PreS-O can be easily adapted by replacing RBD from Wuhan Hu-1 with further evolving Omicron (**Supplementary Figure S4**) or even by RBDs from other newly emerging SARS-CoV-2 variants.

## Data Availability

The original contributions presented in the study are included in the article/**Supplementary Material**. Further inquiries can be directed to the corresponding author.
