# Supplementary material for: Fusion protein-based COVID-19 vaccines exemplified by a chimeric vaccine based on a single fusion protein (W-PreS-O)
Source: Front Immunol. 2025 Jan 28;16:1452814. doi: 10.3389/fimmu.2025.1452814 (PMC11811753; doi:10.3389/fimmu.2025.1452814)
Supplement: Supplementary file 1 [file DataSheet1.docx]

**Supplementary Data**

**Fusion protein-based COVID-19 vaccines exemplified by a chimeric vaccine based on a single fusion protein (W-PreS-O)**

Pia Gattinger ^a†^, Luibov I. Kozlovskaya ^b†^, Alexander S. Lunin ^b^, Olga S. Gancharova ^b^, Dina I. Sirazova ^b,c^, Vasiliy D. Apolokhov ^b^, Egor S. Chekina ^b^, Ilya V. Gordeychuk ^b,c^, Alexander V. Karaulov ^d,f^, Rudolf Valenta ^a,d,e,f*^and Aydar A. Ishmukhametov ^b,c*^

^a^ Medical University of Vienna, Center for Pathophysiology, Infectiology and Immunology, Department of Pathophysiology and Allergy Research, Division of Immunopathology, 1090 Vienna, Austria;

^b^ Chumakov Federal Scientific Center for Research and Development of Immune-and-Biological Products of Russian Academy of Sciences (Institute of Poliomyelitis), 108819 Moscow, Russia

^c^ Sechenov First Moscow State Medical University, Institute for Translational Medicine and

Biotechnology, 119048 Moscow, Russia

^d^ Sechenov First Moscow State Medical University, Laboratory for Immunopathology, Department of Clinical Immunology and Allergology, 119048 Moscow, Russia

^e^ Life Improvement by Future Technologies (LIFT) Center, 143025 Moscow, Russia.

^f^ Center for Molecular Allergology, Karl Landsteiner University of Health Sciences, 3500 Krems, Austria;

^†^ These authors contributed equally to this work

^#^ These authors contributed equally to this work

* Corresponding author

Rudolf Valenta

Department of Pathophysiology and Allergy Research

Medical University of Vienna

Waehringer Guertel 18-20

A-1090 Vienna, Austria

Tel: +43-1-40400-50420

Tel: +43-69912570519

Fax: +43-1-40400-51300

E-mail: rudolf.valenta@meduniwien.ac.at

1.1. Materials and Methods

**1.1. Cells and viruses**

The Vero cell line was obtained from Biologicals, World Health Organization, Switzerland, and maintained in Dulbecco's Modified Eagle Medium (DMEM, Chumakov FSC R&D IBP RAS, Moscow, Russia), supplemented with 5% fetal bovine serum (FBS) (Gibco, Thermo Fisher, Waltham, MA, USA), streptomycin (0.1 mg/ml), and penicillin (100 units/ml) (both PanEco, Moscow, Russia).

The SARS-CoV-2 variant Omicron strain 7995o (Pango lineage BA.1-like, GISAID EPI_ISL_9613539) was stored as cell culture supernatant obtained from infected cells at –70 °C.

**1.2. Immunization of animals and infection model**

In a previous study we found that W-PreS-O was more effective in inducing Omicron-neutralizing antibodies than O-PreS-O, W-PreS-W, and a mix of O-PreS-O and W-PreS-W (Gattinger et al., 2024). Therefore, W-PreS-O was selected as fusion protein-based candidate vaccine to be tested in the Syrian hamster model for its ability to induce *in vivo* protective antibody responses. This study was designed to compare the aluminum hydroxide-adsorbed W-PreS-O vaccine with an adequate placebo preparation (i.e., aluminum hydroxide alone). Recombinant fusion protein W-PreS-O (Figure S1A), comprising Hepatitis B virus (HBV) PreS with a N-terminal RBD from SARS-CoV-2 strain Hu-1 (Genbank accession Nr.: QHD43416.1) and a C-terminal RBD from SARS-CoV-2 variant Omicron (Pango B.1.1.529), was expressed in HEK293F cells (ThermoFisher, Waltham, MA, USA) and purified as previously described (Gattinger et al., 2022, Gattinger et al., 2024). W-PreS-O was adsorbed onto aluminum hydroxide (SERVA Electrophoresis, Heidelberg, Germany) as described (Gattinger et al., 2024). The final doses of the 150 µl vaccine formulation contained 40 µg W-PreS-O, 0.6 mg/ml aluminum hydroxide in 10 mM NaH_2_PO_4_, 0.9% NaCl, pH 7.2. Formulations containing 150 µl per dose of 0.6 mg/ml aluminum hydroxide in 10 mM NaH_2_PO_4_, 0.9% NaCl, pH 7.2, served as the placebo control.

Outbred Syrian hamsters (*Mesocricetus auratus*), males, 35–40 g (4 weeks old), were purchased from the Scientific Center of Biomedical Technologies, branch Stolbovaya, Russia. Randomization of animals was performed by weight into groups: intact (*n* = 4), placebo (*n* = 16), and vaccine (*n* = 16). The placebo and vaccine group animals were immunized intramuscularly (in the upper third of the hind limb) three times at 21-day intervals with 150 µl of the placebo or vaccine preparation per dose (Figure S1B). The placebo and vaccine group animals were infected intra-nasally with 10^4^ TCID_50_ (25 µL into each nostril) of the SARS-CoV-2 variant Omicron (strain 7995o) 21 days after the third vaccination. The strain used for infection (i.e., strain 7995o) differed in only one conservative exchange in the RBD from the BA.1 variant used for the vaccine design (Supplemental Figure S3). The amino acid sequence of the complete W-PreS-O vaccine antigen can be found in Supplemental Figure S2.

The animals were observed daily, weighed change was determined as in (Hajnik et al., 2022, Hajnik et al., 2024) [(weight day_0_-weight day_n_)/weight day_0_*100*-1], and oropharyngeal swabs were collected. Eight animals from each infected group were euthanized on day 3 and day 7 post-infection, respectively (d.p.i.) (Figure S1B). The lungs and nasal cavities were collected and frozen for further investigation. Blood samples were collected before each vaccination and at each euthanasia point; serum samples were stored at -20 °C for neutralization tests. Since the main goal of this study was to compare the vaccinated with the non-vaccinated (i.e., placebo-treated) animals, the animals from the “intact group” were neither immunized nor infected and served ~~as~~ only as controls to ensure that the infection occurred in a specific manner.

The animal study protocol was approved by the Ethics Committee of the Chumakov FSC R&D IBP RAS (Protocol 190722-2, dated 19.06.22). The animals were maintained in accordance with the Directive of the European Parliament and of the Council 2010/63/ EU dated September 22, 2010 on the protection of animals used for scientific purposes. Before euthanasia, animals were anesthetized with intramuscular administration of "Zoletil100" 20 mg/kg and "Xyla" 5 mg/kg intramuscularly. Euthanasia itself was performed by threefold anesthetics overdose. Confirmation of death was carried out by recording the absence of heartbeat, respiratory movements and decapitation.

**1.3. Determination of viral loads**

Homogenization of organs with physiological saline buffer was performed using a TissueLyser (Qiagen, Venlo, Netherlands). For lung tissue 0.02g per sample were used for RT-PCR. Swabs and organ suspensions were examined for the presence of viral RNA using POLYVIR SARS-CoV-2 (Lytech, Moscow, Russia), according to the manufacturer’s protocolQuantification of viral RNA was normalized per weight of organ. Semi-quantitative results are given as cycle threshold (Ct values). The viral load in the lung suspensions was determined via titration in Vero cells. For this purpose, ten-fold dilutions of organ suspensions were prepared in DMEM (Chumakov FSC R&D IBP RAS, Moscow, Russia) and added to confluent Vero cell monolayers on 24-well plates. After 1 h of incubation at 37 °C for virus adsorption, the wells were overlaid with 1.5% methylcellulose (Sigma, Burlington, MA, USA) on DMEM containing 2% FBS. After 5 days of incubation at 37 °C, the cells were fixed with 96% ethanol and stained with 0.4% crystal violet. Plaques were counted visually, and the virus titers were expressed as plaque forming units (PFU) per gram of infected tissue (logPFU/g).

**1.4. Virus neutralization test**

Serum samples were diluted (two-fold, starting from 1:8) in DMEM and mixed with equal volumes of virus, containing in total 50–200 CCID_50_ per well. After an incubation period of 1 hour at 37 °C, the serum–virus mixtures were added, in two replicates each, to confluent Vero cell monolayers. As controls, non-immune and standard immune control sera were used, and a virus dose titration was performed. The controls were performed in parallel to the serum samples derived from the animals. After five days of incubation at 37 °C, the cytopathic effect (CPE) was visually assessed via light microscopy. Neutralizing antibody titers were calculated according to Kärber (Kärber, 1931). Results of <1:8 were considered negative (0).

**1.5. Histological examination**

Lung samples were fixed for 48 hours at room temperature in 10% neutral buffered formalin, and after dehydration in isopropyl alcohol, the samples were embedded in paraffin medium. Subsequently, two micrometer-thick cross-sections from all lung lobes (two sections for each tissue block) were obtained, and representative sections were stained with Mayer's hematoxylin and hydroalcoholic eosin (both Biovitrum, Moscow, Russia) using Leica ST5010 AXL autostainer (Leica, Wetzlar, Germany). Histological preparations were converted into a digital format using a KF-PRO-400 histological scanner (KFBio, Zhejiang, China). The resulting digital versions of the histological preparations were examined using a Leica Aperio Imagescope (Leica, Wetzlar, Germany) and K-Viewer software (IQM, Oslo, Norway). Representative microphotographs were obtained using the snapshot tool in the indicated software. The severity of the pathological process (pneumonia signs, including inflammation, presence of fibrinous exudate in the alveoli, loss of alveolar pattern) in the lungs of each animal was assessed according to the pneumonia intensity score (0–4), where the signs of pathological processes were rated as follows: 0—are not visible, 1—mildly pronounced, 2—moderately pronounced, 3—sharply pronounced, and 4—extremely pronounced. We also visually assessed the area of the lung tissue involvement with pathological processes (area of lung lesions) as the pneumonia area score (0–4), where 0—no changes or the lesion involved less than 10% of the area; 1—lesion involved 10–25% of the area; 2—lesion involved 25–50% of the area; 3—lesion involved 50–75% of the area; and 4—lesion involved 75–100% of the area. The results of the semi-quantitative scoring, i.e., pneumonia area score and pneumonia intensity score, were multiplied to obtain the pneumonia index for each individual lung slide (two per animal).

**1.6. Statistical analysis**

Differences in antibody titers and Ct-values were determined using the Mann–Whitney test, and the weight curves were compared using an ANOVA with OriginPro Version 8 (OriginLab, Northampton, MA, USA). P values < 0.05 were considered significant.

**References**

GATTINGER, P., KRATZER, B., SEHGAL, A. N. A., OHRADANOVA-REPIC, A., GEBETSBERGER, L., TAJTI, G., FOCKE-TEJKL, M., SCHAAR, M., FUHRMANN, V., PETROWITSCH, L., KELLER, W., HÖGLER, S., STOCKINGER, H., PICKL, W. F. & VALENTA, R. 2024. Vaccine Based on Recombinant Fusion Protein Combining Hepatitis B Virus PreS with SARS-CoV-2 Wild-Type- and Omicron-Derived Receptor Binding Domain Strongly Induces Omicron-Neutralizing Antibodies in a Murine Model. *Vaccines (Basel),* 12.

GATTINGER, P., KRATZER, B., TULAEVA, I., NIESPODZIANA, K., OHRADANOVA-REPIC, A., GEBETSBERGER, L., BOROCHOVA, K., GARNER-SPITZER, E., TRAPIN, D., HOFER, G., KELLER, W., BAUMGARTNER, I., TANCEVSKI, I., KHAITOV, M., KARAULOV, A., STOCKINGER, H., WIEDERMANN, U., PICKL, W. F. & VALENTA, R. 2022. Vaccine based on folded receptor binding domain-PreS fusion protein with potential to induce sterilizing immunity to SARS-CoV-2 variants. *Allergy,* 77**,** 2431-2445.

HAJNIK, R. L., PLANTE, J. A., LIANG, Y., ALAMEH, M. G., TANG, J., BONAM, S. R., ZHONG, C., ADAM, A., SCHARTON, D., RAFAEL, G. H., LIU, Y., HAZELL, N. C., SUN, J., SOONG, L., SHI, P. Y., WANG, T., WALKER, D. H., SUN, J., WEISSMAN, D., WEAVER, S. C., PLANTE, K. S. & HU, H. 2022. Dual spike and nucleocapsid mRNA vaccination confer protection against SARS-CoV-2 Omicron and Delta variants in preclinical models. *Sci Transl Med,* 14**,** eabq1945.

HAJNIK, R. L., PLANTE, J. A., REDDY BONAM, S., RAFAEL, G. H., LIANG, Y., HAZELL, N. C., WALKER, J., REYNA, R. A., WALKER, D. H., ALAMEH, M. G., WEISSMAN, D., WEAVER, S. C., PLANTE, K. S. & HU, H. 2024. Broad protection and respiratory immunity of dual mRNA vaccination against SARS-CoV-2 variants. *NPJ Vaccines,* 9**,** 160.

KÄRBER, G. 1931. Beitrag zur kollektiven Behandlung pharmakologischer Reihenversuche. *Naunyn-Schmiedebergs Archiv für experimentelle Pathologie und Pharmakologie,* 162**,** 480-483.

**Supplemental Tables**

**Table S1.** SARS-CoV-2 Omicron neutralizing antibody titers

| **Animal ID** | **Group** | **Neutralizing antibody titers** | | | | | | |
| --- | --- | --- | --- | --- | --- | --- | --- | --- |
|  |  | **day 0** | **day 21** | **day 42** | **day 49** | **day 63** | **day 66**^1^ | **day 70**^2^ |
| 1 | Vaccine | <8 | <8 | <8 | <8 | <8 | <8 | n.a. |
| 2 | Vaccine | <8 | <8 | <8 | **11** | **23** | **45** | n.a. |
| 3 | Vaccine | <8 | <8 | <8 | **16** | **23** | **32** | n.a. |
| 4 | Vaccine | <8 | <8 | <8 | **91** | **181** | **91** | n.a. |
| 5 | Vaccine | <8 | <8 | <8 | 8 | **16** | **16** | n.a. |
| 6 | Vaccine | <8 | <8 | <8 | **11** | **32** | **23** | n.a. |
| 7 | Vaccine | <8 | <8 | <8 | **91** | **256** | **128** | n.a. |
| 8 | Vaccine | <8 | <8 | <8 | <8 | <8 | **11** | n.a. |
| 9 | Vaccine | <8 | <8 | <8 | <8 | <8 | n.a. | **>1024** |
| 10 | Vaccine | <8 | <8 | <8 | **128** | **512** | n.a. | **362** |
| 11 | Vaccine | <8 | <8 | <8 | **91** | **91** | n.a. | **1024** |
| 12 | Vaccine | <8 | <8 | <8 | <8 | **11** | n.a. | **>1024** |
| 13 | Vaccine | <8 | <8 | <8 | **23** | **45** | n.a. | **1024** |
| 14 | Vaccine | <8 | <8 | <8 | **11** | **32** | n.a. | **>1024** |
| 15 | Vaccine | <8 | <8 | <8 | **16** | **64** | n.a. | **1024** |
| 16 | Vaccine | <8 | <8 | <8 | <8 | **8** | n.a. | **>1024** |
| 17 | Placebo | <8 | <8 | <8 | <8 | <8 | <8 | n.a. |
| 18 | Placebo | <8 | <8 | <8 | <8 | <8 | <8 | n.a. |
| 19 | Placebo | <8 | <8 | <8 | <8 | <8 | <8 | n.a. |
| 20 | Placebo | <8 | <8 | <8 | <8 | <8 | <8 | n.a. |
| 21 | Placebo | <8 | <8 | <8 | <8 | <8 | <8 | n.a. |
| 22 | Placebo | <8 | <8 | <8 | <8 | <8 | <8 | n.a. |
| 23 | Placebo | <8 | <8 | <8 | <8 | <8 | <8 | n.a. |
| 24 | Placebo | <8 | <8 | <8 | <8 | <8 | <8 | n.a. |
| 25 | Placebo | <8 | <8 | <8 | <8 | <8 | n.a. | **512** |
| 26 | Placebo | <8 | <8 | <8 | <8 | <8 | n.a. | **724** |
| 27 | Placebo | <8 | <8 | <8 | <8 | <8 | n.a. | **724** |
| 28 | Placebo | <8 | <8 | <8 | <8 | <8 | n.a. | **724** |
| 29 | Placebo | <8 | <8 | <8 | <8 | <8 | n.a. | **724** |
| 30 | Placebo | <8 | <8 | <8 | <8 | <8 | n.a. | **724** |
| 31 | Placebo | <8 | <8 | <8 | <8 | <8 | n.a. | **1024** |
| 32 | Placebo | <8 | <8 | <8 | <8 | <8 | n.a. | **1024** |
| 33 | Intact | <8 | <8 | <8 | <8 | <8 | <8 | n.a. |
| 34 | Intact | <8 | <8 | <8 | <8 | <8 | <8 | n.a. |
| 35 | Intact | <8 | <8 | <8 | <8 | <8 | n.a. | <8 |
| 36 | Intact | <8 | <8 | <8 | <8 | <8 | n.a. | <8 |

n.a. = not applicable , ^1^ 3 days after infection, ^2^ 7 days after infection

**Table S2.** Histological examination of lungs of experimental animals

| **Animal ID** | **Group** | **Day of euthanasia** | **Pneumonia intensity score (0-4) per slide^1,2^** | **Pneumonia area score (0-4) per slide^3^** | **Pneumonia Index (0-16) per slide**^4^ |
| --- | --- | --- | --- | --- | --- |
| 1 | Vaccine | 3 | 0 | 0 | 0 |
| 2 | Vaccine | 3 | 0 | 0 | 0 |
|  |  |  | 0 | 0 | 0 |
| 3 | Vaccine | 3 | 0 | 0 | 0 |
| 4 | Vaccine | 3 | 0 | 0 | 0 |
| 5 | Vaccine | 3 | 0 | 0 | 0 |
|  |  |  | 0 | 0 | 0 |
| 6 | Vaccine | 3 | 0 | 0 | 0 |
|  |  |  | 0 | 0 | 0 |
| 7 | Vaccine | 3 | 0 | 0 | 0 |
|  |  |  | 0 | 0 | 0 |
| 8 | Vaccine | 3 | 0 | 0 | 0 |
|  |  |  | 0 | 0 | 0 |
| 9 | Vaccine | 7 | 1 | 1 | 1 |
|  |  |  | 1 | 2 | 2 |
| 10 | Vaccine | 7 | 0 | 0 | 0 |
|  |  |  | 1 | 1 | 1 |
| 11 | Vaccine | 7 | 0 | 0 | 0 |
|  |  |  | 1 | 1 | 1 |
| 12 | Vaccine | 7 | 0 | 0 | 0 |
|  |  |  | 1 | 1 | 1 |
| 13 | Vaccine | 7 | 0 | 0 | 0 |
|  |  |  | 0 | 0 | 0 |
| 14 | Vaccine | 7 | 1 | 1 | 1 |
|  |  |  | 1 | 2 | 2 |
| 15 | Vaccine | 7 | 1 | 2 | 2 |
|  |  |  | 1 | 2 | 2 |
| 16 | Vaccine | 7 | 0 | 0 | 0 |
|  |  |  | 1 | 2 | 2 |
| 17 | Placebo | 3 | 0 | 0 | 0 |
|  |  |  | 0 | 0 | 0 |
| 18 | Placebo | 3 | 2 | 2 | 4 |
|  |  |  | 2 | 2 | 4 |
| 19 | Placebo | 3 | 1 | 1 | 1 |
|  |  |  | 1 | 2 | 2 |
| 20 | Placebo | 3 | 1 | 1 | 1 |
|  |  |  | 3 | 2 | 6 |
| 21 | Placebo | 3 | 1 | 3 | 3 |
|  |  |  | 1 | 2 | 2 |
| 22 | Placebo | 3 | 1 | 3 | 3 |
|  |  |  | 1 | 2 | 2 |
| 23 | Placebo | 3 | 2 | 2 | 4 |
|  |  |  | 2 | 2 | 4 |
| 24 | Placebo | 3 | 0 | 0 | 0 |
|  |  |  | 1 | 2 | 2 |
| 25 | Placebo | 7 | 0 | 0 | 0 |
| 25  26 | Placebo  Placebo | 7  7 | 0 | 0 | 0 |
|  |  |  | 0 | 0 | 0 |
| 26  27 | Placebo  Placebo | 7  7 | 0 | 0 | 0 |
|  |  |  | 2 | 2 | 4 |
| 27  28 | Placebo  Placebo | 7  7 | 2 | 2 | 4 |
|  |  |  | 0 | 0 | 0 |
| 28  29 | Placebo  Placebo | 7  7 | 0 | 0 | 0 |
|  |  |  | 2 | 3 | 6 |
| 29  30 | Placebo  Placebo | 7  7 | 2 | 3 | 6 |
|  |  |  | 3 | 2 | 6 |
| 30  31 | Placebo  Placebo | 7  7 | 3 | 2 | 6 |
|  |  |  | 3 | 3 | 9 |
| 31  32 | Placebo  Placebo | 7  7 | 3 | 3 | 9 |
|  |  |  | 3 | 3 | 9 |
| 32  33 | Placebo  Intact | 7  3 | 3 | 3 | 9 |
|  |  |  | 0 | 0 | 0 |
| 33  34 | Intact  Intact | 3  3 | 0 | 0 | 0 |
|  |  |  | 0 | 0 | 0 |
| 34  35 | Intact  Intact | 3  7 | 0 | 0 | 0 |
|  |  |  | 0 | 0 | 0 |
| 35  36 | Intact  Intact | 7  7 | 0 | 0 | 0 |
|  |  |  | 0 | 0 | 0 |
| 36 | Intact | 7 | 0 | 0 | 0 |
|  |  |  |  |  |  |

^1^ Two slides per lung per animal were investigated

^2^ Pneumonia intensity score (0-4) describes the intensity of pneumonia signs (inflammation, characteristic hemorrhagic component, presence of fibrinous exudate in the alveoli with loss of alveolar pattern), where these pathological processes were: 0 – not visible, 1 – mildly pronounced, 2 – moderately pronounced, 3 – sharply pronounced, 4 – extremely pronounced.

^3^ Pneumonia area score (0-4) is a visual assessment of the lung area affected by the pathological process (lesion), where: 0 – no changes or the lesion involves less than 10% of the area; 1 – lesion involves 10-25% of the area; 2 – lesion involves 25-50% of the area; 3 – lesion involves 50-75% of the area; 4 – lesion involves 75-100% of the area.

^4^ Pneumonia index is a multiplication of the semi-quantitative Pneumonia intensity score and Pneumonia area score (for each slide, 2 per animal)

**Supplemental Figures**

**~~
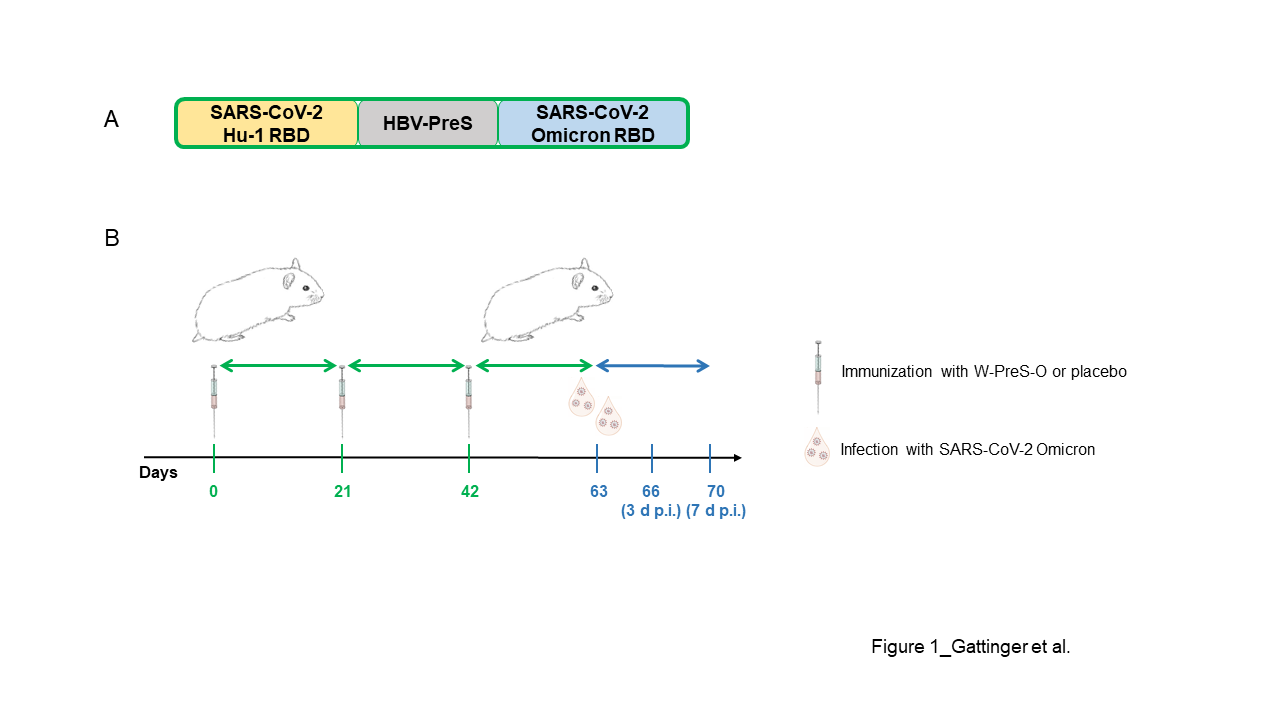
~~**

**Figure S1.** Schematic representation of **(A)** the recombinant fusion protein W-PreS-O, comprising RBDs from SARS-CoV- 2 Hu-1 (yellow) and Omicron BA.1 (blue), fused to the HBV-surface anti-gen PreS (grey). **(B)** Immunization schedule (W-PreS-O: n=16; Placebo: n=16) and infection model for the Syrian hamsters. Days post-infection = d.p.i.

MYRMQLLSCIALSLALVTNSPNITNLCPFGEVFNATRFASVYAWNRKRISNCVADYSVLYNSASFSTFKCYGVSPTKLNDLCFTNVYADSFVIRGDEVRQIAPGQTGKIADYNYKLPDDFTGCVIAWNSNNLDSKVGGNYNYLYRLFRKSNLKPFERDISTEIYQAGSTPCNGVEGFNCYFPLQSYGFQPTNGVGYQPYRVVVLSFELLHAPGGWSSKPRKGMGTNLSVPNPLGFFPDHQLDPAFGANSNNPDWDFNPIKDHWPAANQVGVGAFGPGLTPPHGGILGWSPQAQGILTTVSTIPPPASTNRQSGRQPTPISPPLRDSHPQAMQWNSTAFHQALQDPRVRGLYFPAGGSSSGTVNPAPNIASHISSISARTGDPVTNPNITNLCPFDEVFNATRFASVYAWNRKRISNCVADYSVLYNLAPFFTFKCYGVSPTKLNDLCFTNVYADSFVIRGDEVRQIAPGQTGNIADYNYKLPDDFTGCVIAWNSNKLDSKVSGNYNYLYRLFRKSNLKPFERDISTEIYQAGNKPCNGVAGFNCYFPLRSYSFRPTYGVGHQPYRVVVLSFELLHAP*HHHHHH*

**Figure S2.** Amino acid sequence of fusion protein W-PreS-O. Black and underlined sequence represents the IL-2 signal peptide which is cleaved, yellow sequence: the RBD from Hu-1 (Genbank accession Nr.: QHD43416.1), grey: sequence of HBV-derived PreS, blue sequence RBD-Omicron BA.1 (PANGO B.1.1.529) and black italic the hexa-histidine tag.

HU-1 330 PNITNLCPFGEVFNATRFASVYAWNRKRISNCVADYSVLYNSASFSTFKCYGVSPTKLND 389

BA.1 330 .........D...............................L.P.F.............. 389

7995o 330 .........D......K........................L.P.F.............. 389

HU-1 390 LCFTNVYADSFVIRGDEVRQIAPGQTGKIADYNYKLPDDFTGCVIAWNSNNLDSKVGGNY 449

BA.1 390 ...........................N......................K.....S... 449

7995o 390 ...........................N......................K.....S... 449

HU-1 450 NYLYRLFRKSNLKPFERDISTEIYQAGSTPCNGVEGFNCYFPLQSYGFQPTNGVGYQPYR 509

BA.1 450 ...........................NK.....A........R..S.R..Y...H.... 509

7995o 450 ...........................NK.....A........R..S.R..Y...H.... 509

HU-1 510 VVVLSFELLHAP 521

BA.1 510 ............ 521

7995o 510 ............ 521

**Figure S3.** Sequence alignment of SARS-CoV-2 wild type RBD (HU-1, Genbank accession Nr.: QHD43416.1) with the amino acid sequences from RBD Omicron variant BA.1 (PANGO B.1.1.529) and Omicron BA.1-like sub variant strain 7995o (Pango lineage BA.1-like, GISAID EPI_ISL_9613539) . Different amino acids are indicated, identical amino acids are indicated by dots and positions in the S protein are given on the margins.

BA1 330 PNITNLCPFDEVFNATRFASVYAWNRKRISNCVADYSVLYNLAPFFTFKCYGVSPTKLND 389

BA4/5 330 .........................................F.................. 389

XBB1.5 330 .........H......T.....................I..F.................. 389

XBB1.16 330 .........H......T.....................I..F.................. 389

BA2 330 ............................................................ 389

JN.1 330 ................T........................................... 389

KP.2 330 ................T........................................... 389

KP.3 330 ................T........................................... 389

BA1 390 LCFTNVYADSFVIRGDEVRQIAPGQTGNIADYNYKLPDDFTGCVIAWNSNKLDSKVSGNY 449

BA4/5 390 ...............N..S......................................... 449

XBB1.5 390 ...............N..S....................................P.... 449

XBB1.16 390 ...............N..S....................................P.... 449

BA2 390 ..................S......................................... 449

JN.1 390 ..................S......................................... 449

KP.2 390 ..................S......................................... 449

KP.3 390 ..................S......................................... 449

BA1 450 NYLYRLFRKSNLKPFERDISTEIYQAGNKPCNGVAGFNCYFPLRSYSFRPTYGVGHQPYR 509

BA4/5 450 ..R.................................V....................... 509

XBB1.5 450 ..........K.........................P...S................... 509

XBB1.16 450 ..........K.................P.......P...S................... 509

BA2 450 ............................................................ 509

JN.1 450 ......L..................................................... 509

KP.2 450 ......L....................................E................ 509

KP.3 450 ......L....................................E................ 509

BA1 510 VVVLSFELLHAP 521

BA4/5 510 ............ 521

XBB1.5 510 ............ 521

XBB1.16 510 ............ 521

BA2 510 ............ 521

JN.1 510 ............ 521

KP.2 510 ............ 521

KP.3 510 ............ 521

**Figure S4.** Sequence alignment of SARS-CoV-2 RBD BA.1 with RBD with the amino acid sequences from RBD Omicron variants. Different amino acids are indicated, identical amino acids are indicated by dots and positions in the S protein are given on the margins. The sequences were colored to illustrate features of the amino acids (light red = acidic hydrophilic, yellow= neutral, light green=basic hydrophilic, light blue=hydrophobic).

**
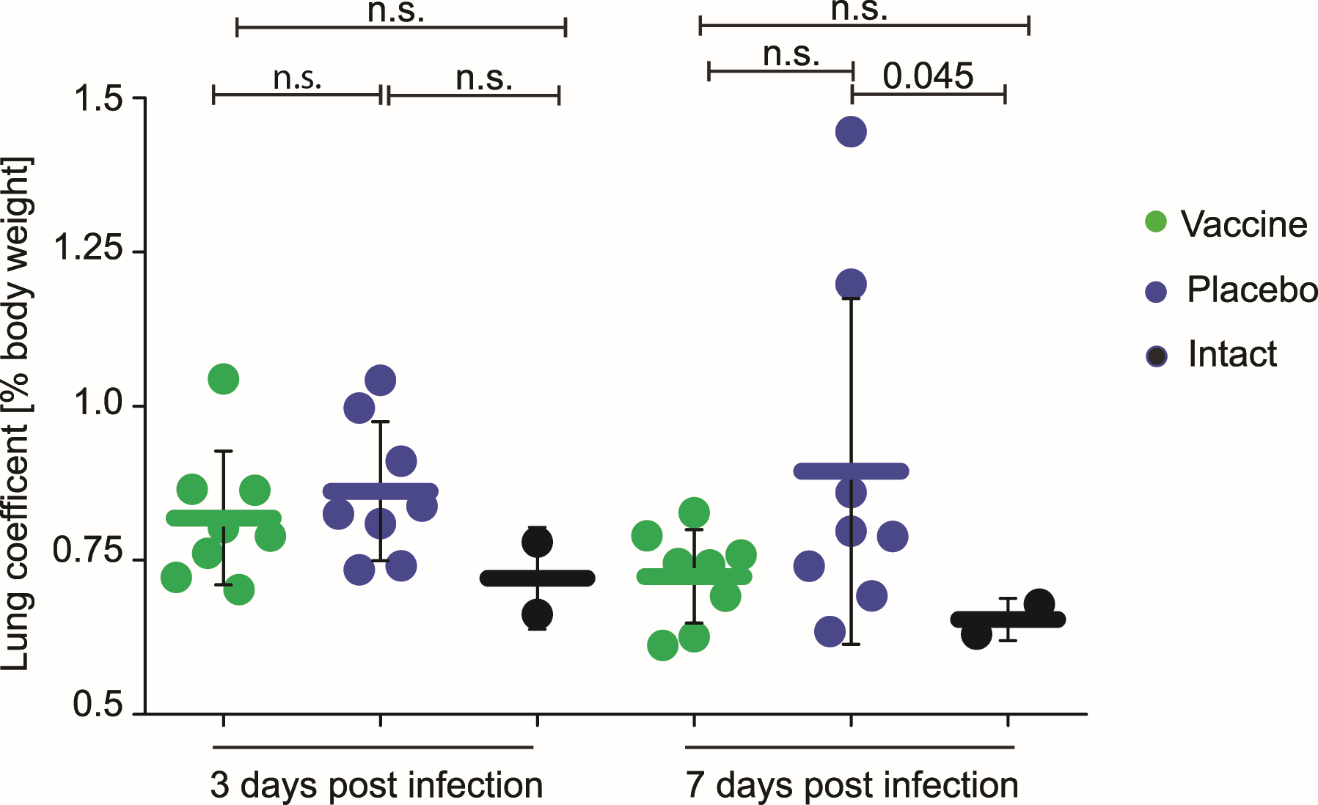
**

**Figure S5.** Severity of lung lesions in the different groups of infected animals. Pneumonia-caused lung edema is presented as the weight coefficient of the total body weight (y-axis).
